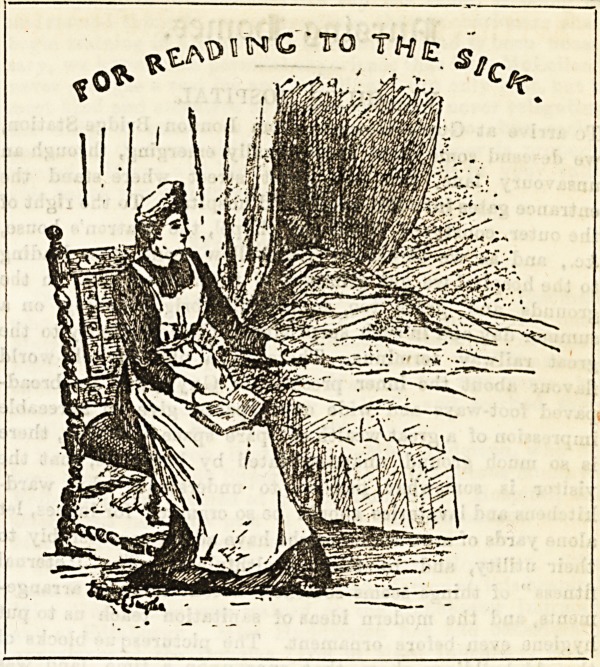# The Hospital Nursing Supplement

**Published:** 1892-10-08

**Authors:** 


### "The Hospital" Nursing Mirror.

Being the Extra Nursing Supplement of "The Hospital" Newspaper.

[Contributions for this Supplement should be addressed to the Editor, The Hospital, 140, Strand, London, W.C., and should have the word "Nursing" plainly written in left-hand top corner of the envelope.]

### En Passant.

NEWS FROM NICOSIA.—Miss Amy M. Potter who went out to Cyprus two years ago in connection with the Cyprus Society, and was afterwards appointed Lady Superintendent of the Government Hospital, Nicosia, has resigned her appointment on the occasion of her marriage.

BRABAZON HOME OF COMFORT, REIGATE.—This Home was founded by the Countess of Meath for G.F.S. members, and has been of inestimable service to these useful and deserving girls. They get the long care and treatment necessary to ensure their restoration to complete health. The Home is out of debt, and has a small balance in hand, and further subscriptions are desired to establish it on a firm financial basis. Gifts of books, shawls, nightgowns, pillows, wool for knitting, &c., are most acceptable.

MIDLETON GUARDIANS AND THE NUNS.—The nuns have nursed the sick paupers in Midleton Workhouse for six years, and now the *Freeman's Journal* reports that they have resigned their respective positions of Matron and nurses. They do not appear to have given a reason for their action, but after a long discussion by the members of the committee it was unanimously agreed not to accept their resignation. Their services appear to have been highly valued, and the Board evidently does not intend to relinquish them until a fortnight has elapsed, when the matter is to be again under consideration.

CHILDREN'S HOSPITALS AND CONVALESCENT HOMES.—Every children's ward in an adult hospital, and every institution specially devoted to the treatment of children ought to possess a country or seaside branch. The advantages of such an adjunct needs no comment, but comparatively few are at present in existence. Aberdeen Children's Hospital is one of the fortunate minority, for it owns a country branch which is open during the summer months, and is staffed by a couple of nurses from headquarters. "By this plan not only do the children benefit, but the nurses are greatly the better in every way for their residence in the country," writes an Aberdeen friend.

AN OUT-PATIENT DEPARTMENT.—There was a most amusing description in the *Pall Mall* of October 1st of a scene in the physician's room in the out-patient department of some London hospital. The writer has given his character-sketches with a keen realism which appeals successfully to all who know the daily routine of these busy spots. The patients so graphically represented are true to the life, and a kindly satire lays bare to the reader many traits which only hospital workers have hitherto grasped. What out-patient nurse did not smile at the mother who refused to leave her child in for the operation, which is his sole hope of life, and then demanded medicine, and "wants to know" what 'orspitals are for, if they don't give medicine? The mother who sits amongst other parents with a child covered with measles rash is an equally familiar and impenitent transgressor. The child who uses bad language, which his mother describes as his form of shyness with strangers! is delightfully natural, and his outrageous behaviour whilst she remains on the scene is well contrasted with the change when she retires in favour of the nurse, and "Freddy resigns himself to his fate and becomes perfectly docile, with the pleasing result that nothing is found the matter with him

NURSES IN WORKHOUSES.—We learn from the Plymouth papers that the Plympton Guardians have just appointed a nurse. There were only two applicants for the post, which, as Dr. Aldridge remarked, "was a sufficient reflection on the resolution of the Board not to appoint a properly-trained hospital nurse at a fair salary." Evidently this is not the first time the attention of the Guardians has been called to the importance of a qualified person being secured, but the "fair salary" is apparently considered as unnecessary for the attendant, as skilled nursing is for the unfortunate sick paupers at Plympton.

AN UNGRATEFUL CONVALESCENT.—This is the title which the *Western Mail* aptly applies to the writer of a letter in the *Standard* on "The Cost of Trained Nurses." This thankless person has evidently safely recovered from a dangerous illness, and during it he was tended by two trained nurses. Surely it would be well to deduct the item of restored health from the fees so grudgingly paid for skilled labour. Has "the ungrateful convalescent" quite forgotten what he owes to patient care and intelligent nursing, or is he equally unwilling to pay his doctor? Skilled labour surely merits fair pay, and the help which is demanded by the sick man should be cheerfully remunerated by the convalescent.

TYN-Y-COED CONVALESCENT HOME.—The September number of "Forward," the journal of the Birmingham Hospital Saturday Fund, contains an interesting account of the Convalescent Home, which is evidently working most satisfactorily. A late patient gives a detailed and pleasant description of his stay there, and speaks warmly of the general arrangements and "the large liberty allowed in things not essential; to treat men as children seems to me utterly ridiculous, and I, for my part, beg to thank the authorities here for the trust they reposed in us at all times. I do not think they found their confidence misplaced." The ex-patient also praises the "admirable meals" supplied to the visitors, and speaks of the good quality and the great variety of the food.

TOTTENHAM TEMPORARY FEVER HOSPITAL.—On October 10th it is proposed to open this new building for the reception of patients. A good supply of nurses is already forthcoming, but a few additional staff nurses will probably be required later on. The "Superior" of the All Saints' Sisterhood is organising the nursing arrangements, and she has no easy task to accomplish in so new a structure. The cubicles are conveniently arranged and adequately furnished, both for the nursing staff and the ward servants. The wards themselves are light and airy, but we are sorry to think of restless fever patients condemned to bedsteads which have not the slightest semblance of a foot-rail. It can add very little to the expense, but it enormously increases the comfort of a bed to have some controlling support for the mattress and clothes; certainly sick people should be indulged in this small but important matter. The baths are of convenient shape and size, which is more than can be said of the slop-sinks in the lavatories, which appear expressly designed not to answer their nominal purpose. This is very shortsighted management, and we did not expect to see such an important nursing detail overlooked in these days of advanced sanitation. The best pattern slop-sink can be seen at King's or the London Hospitals, and at the Liverpool Royal Infirmary.

### Lectures for Asylum Attendants.

By William Harding, M.B.

### IV.—WARMTH.

This subject is a very important one in the management of the insane. Many of them are peculiarly susceptible to the influence of changes in the temperature, and are markedly affected by even a slight fall in the thermometer. There is no doubt that the popular idea of the many evils which owe their origin to "catching a cold" is very exaggerated. That exposure to cold does bring on some forms of disease is, however, certain, and the more debilitated the individual, the more likelihood there is of his falling a victim. The young, the feeble, and the aged require a higher temperature and warmer clothing than are sufficient for an adult in fair health. Especially amongst the demented classes (or those suffering from mental weakness) must this point be carefully attended to. Their nervous force is small, and their circulation is often weak. Cold and blue extremities are common among them. Chilblains are easily produced, and not readily got rid of. In such cases bronchial catarrh, diarrhœa, and low forms of inflammation are the not infrequent results of exposure to chills. In one class of cases, that of acute dementia, the question of warmth is almost as important as that of food, and the patients will often with advantage bear a temperature which seems unpleasantly warm to the other occupants of the room. Such patients are generally much brighter in summer than in winter. It will be noticed, too, that epileptics do best who occupy beds in the warmest part of the dormitory.

In cold weather the problem of keeping a ward containing dirty cases up to the proper temperature, and at the same time sweet and well ventilated, is one not always easy of solution. More especially is it difficult when the wards are full of the very demented. These, the very people who require most warmth, are unable to help themselves, and are too dull and stupid even to complain. They will plant themselves in a direct draught with unfastened garments, and although they may become pinched and blue with cold, have not enough intelligence to shift their position. They are thus in a peculiar manner dependent upon the thoughtfulness of the nurse. If it be necessary that the part of the ward in which they are seated should be blown through, they should be removed out of the cold draught until this is done. The early morning, before the sun has gained much power, is the time when the heating arrangements generally fail. Patients should not be brought from warm beds to sit and shiver in the day rooms until they are warmed up. The day rooms should be raised to a temperature of 55 deg. F. before the patients are allowed to enter them. If all the lunatics were able to employ themselves in ward work, and thus keep themselves warm, the case might not be so bad, but such as can do this are in some of the wards but too few in number. It is a painful sight in the early morning to see the poor demented creatures sitting with chilled extremities and blue noses, what little intellect they have almost destroyed by the cold.

Where the heating arrangements consist of hot-air or steam-pipes they are out of the nurses' hands. These are safer, and in many respects more convenient, than stoves and open fires. For dormitories not under constant supervision, the latter are, of course, out of the question. But for day rooms, where nurses are constantly present, open fires are more home like, and are to be preferred. In large rooms, of course, they have often to be combined with some other method. If stoves are used, the night nurses should see that they are well stoked up on their last rounds, so as to warm up the corridors before they are occupied. The fires in the day rooms should also be burning brightly before these apartments are tenanted. Though at first sight it is a subject which seems but remotely connected with nursing of the insane, yet lessons in proper stoking and making up of fires should form part of every nurse's education. The one extreme of keeping the grate piled right up the chimney is almost as much to be found fault with as the other of neglecting a fire until it is nearly out, and then extinguishing it completely under a shower of small coals. By the former method you run an unnecessary risk of that awful calamity—an asylum on fire. If the grate be kept properly stoked up to the level of the top bar, and the fire be kept bright, the maximum heating effect will be obtained with the minimum expenditure of fuel, and the least risk of any danger.

The clothing of the patients must be closely looked to. If the body be not properly clothed, a great amount of heat is dissipated and lost to its owner. Clothes act as the preservers of the heat produced by the food taken into the system. The nurse should see that her patients have their proper amount of underclothing. Many of them are very apt, if not watched, to lay aside their flannel under-garments. Those lunatics who are just on the border-line between the fortunate few who can, and the unfortunate many who certainly cannot, attend upon themselves, are the ones who are liable to suffer in this way. Some patients appear to have most fanciful objections to wearing flannel garments. These peculiarities should be resisted on the patient's first entrance to the asylum. Like many other bad habits, they become more fixed and difficult to get rid of every day they are indulged in, and a bad practice, which might have been put right with little difficulty at first, may, if allowed to become fixed, develope into a very troublesome and serious business. Other patients again, from ideas connected with their delusions, will give trouble about their clothing.

Those who are allowed to remain wet are in danger of being chilled, owing to the cooling of the body in the evaporation of the urine. This is a very important point for the night nurse to attend to. With but few exceptions, beds constantly wet mean bad nursing. Regular getting up of doubtful cases, and close attention to those known to be liable to this mishap, will do much to shorten the nightly wet and dirty list. A demented lunatic, on a cold night, partially uncovered and in a wet bed, presents a fine target for disease.

It is a sad defect in our asylum construction that the very parts of the building which should have their heating arrangements most perfect are often the worst off in that respect. Very frequently the single rooms occupied by noisy, dirty, and destructive cases are inadequately warmed. The poor lunatic, too excited to remain in bed, tears up her clothing and bedding and then shivers until the night nurse gives her a fresh supply and puts her into bed, probably only to find her as comfortless on her next visit. It would be difficult to have all such cases under constant supervision, and the night nurse who has them under her care cannot perform impossibilities. Surely it is not impossible to devise and to carry out some means of heating single rooms efficiently for the use of this class of lunatics. Feeble and acute cases, who are not so destructive, can be dealt with by putting them into thick flannel-lined combinations, which lace up the back. In these they are kept warm even when restless and out of bed. The temperature of an ordinary ward should be kept about 55 deg. F., and for the more feeble and demented it should be nearer 60 deg. F. The day nurse ought to note the temperature as shown by the thermometer at regular intervals during the day, and enter it in her report sheet. The night nurse also should mark the temperature in the corridors and dormitories during her hours on duty, and show the figures in her report for the night.

When a patient requires bathing, slippers should be placed on her feet, and her body carefully protected from cold in going to and coming from the bath-room.

Before going out for exercise it is the duty of the nurse to see that each patient has her out-of-door garments tidily put on and her boots fastened. The habit of many of throwing off hats, boots, and cloaks will require constant attention. When the day is cold she must not allow the dull and demented members of her flock to sit still and get chilled, but should keep them moving.

### St. Luke's Home, Vancouver.

ONE HUNDRED POUNDS WANTED.

A year has gone since I visited England and returned laden with help for my work. Then followed a visit from Mr. Burdett, and glad indeed I was to welcome him, that he might see for himself we are a veritable working community. Now I venture once again to ask help. We had been working on steadily until small-pox, that dread disease, was brought in by the China boats. First one then another was stricken among the men working on the wharf. There was no hospital, no isolation, and the authorities used tents and employed any labourers. We got so anxious, for we did not know who would be the next victim. The rector went to the City Council and offered our services if we could have a suitable building to nurse in. However, nothing came of it. At last, in the middle of the night, I had a summons to go up to a place called Howe Sound, a few hours by steamer, to nurse a family of ten, all down with small-pox. A young nurse begged to go with me, and off we started, with a police officer and a man to act as guard and see we did not go out. We landed, and found ourselves quite in a forest, the whole family in a single room, and in all stages of the disease. Three cases were confluent, and the neighbours were all afraid to venture within a mile. However, we got through safely and brought all our patients round, although one girl had seemed to be dying hourly. Then my young nurse took small-pox, so we were moved off to a tent where I nursed her. It proved a very slight attack, but out here quarantine regulations are severe, and we were prisoners for fourteen days. When I got back to Vancouver, of course I found all my engagements at a standstill, and patients rather dreading to see me. However, a general scare came, cases cropping up in all directions, traffic in a measure suspended, and business matters also. This has prevented our making any ready-money since our return. Winter is approaching, our expenses must be increased. If I do not receive help, I must either send the four nurses home, or we must work at anything that comes, and close our Home. All the patients we receive indoors are so absolutely helpless—in some cases they will pay us when they earn something—others cannot pay except by doing odd jobs. Yet our household expenses must go on. Fortunately, I own the house, so there is no rent, or else we could not have managed. We have nursed this year about 150 cases, besides doing our own domestic work, therefore it is not from idle women that this plea for help comes. To keep our Home open and assist sick men and women is our wish. The majority of cases are from the old country, they may be brothers or sisters of many Hospital readers, and we endeavour to give them such home comforts during their illnesses as will prevent their feeling lonely and forgotten. We plead for substantial help, being heavily in debt through the standstill caused by the small-pox, which we nursed voluntarily. We also have a mortgage on the property, which must soon be paid. Our expenses are not large, except as regards the patients, for the nurses, if they do not receive money, always get fed. For means to keep the Home open I plead to the rich to send of their wealth, and to our nurses to interest their friends in our plans. I have two nurses, two probationers, and a housekeeper dependent upon me. Any money sent direct to the Home, or paid in to Sister Frances, Bank of Montreal, Vancouver, or Rev. Henry Fiennes-Clinton, Cromwell Rectory, Newark, England, will be gratefully acknowledged.

OUR PATTERN.

In passing through a room where a great many young people were drawing from models, it was interesting to notice how the character of each came out in his or her work. Some went steadily on putting their whole heart into it, following out every line and making their work as perfect as possible; while they, again, were divided into those who did it from pure love, and those who worked from a strong sense of duty. When the two were combined the effect was prodigious. Others tried to improve on the original only to find in the end that it was altogether a failure. The careless ones took no heed, daubed away their time, and had nothing but a blank canvas, or made their strokes at random, so that again and again they were rubbed out, till the paper was a scene of fruitless efforts blurred and spoiled for ever. How much these young people remind us of the way we spend our lives, God has set before us a Perfect Pattern which he bids us copy; but are our love and duty strong enough to make us do so accurately? I fear greatly that we think our Lord's life was too strict, too self-denying for us to attempt to imitate. We would improve on it by turning this way or that to make it easier, but what resemblance is there when we have done so? We cannot bear hunger and thirst and want and pain, and privations of all sorts; and resent the unkindness of friends and the jeers and blows of our enemies, and we will not turn the left cheek when the right has been smitten. So we go out of the way to avoid pain and suffering, and murmur and rebel when we cannot. We return blow for blow to our enemies, and we quarrel with our best friends. And when at last our love to God quickens, and our conscience urges us on to do our duty, what wrong lines there are to rub out! every attempt to do so only makes matters worse; the Master alone can help us. He can and will cleanse our canvas and make it "white as snow" and guide our hands to copy aright. In all model drawings we can only succeed by carefully looking at the patterns, studying every line and curve, and impressing on our minds what it is we have to do. So should we gaze on our Great Pattern, "with long study and great love," till our hearts are full of His perfect beauty. Then we shall take care not to spoil our lives by bearing our sufferings in a wrong spirit. If we cannot see why we have a crook in our lot, why this line which seemed to run so straight should suddenly end, why our strokes are so heavy where we thought they might be light, we will look to our Model, who bore our griefs and carried our sorrows without a murmur, Who went as a lamb to the slaughter, Who when He was reviled, reviled not again. Not in our own strength can we follow His leading, but with the humble faith of childhood, beseeching Him to make us first see aright, and then set diligently to imitate Him who has said, "Learn of me."

### Nursing Homes.

### VI.—GUY'S HOSPITAL.

To arrive at Guy's Hospital from London Bridge Station, we descend some stone steps, finally emerging, through an unsavoury lane, into the quiet street where stand the entrance gates into this quaint old hospital. To the right of the outer courtyard stand the chapel, the Matron's house, &c., and as we as ascend the shallow stone steps leading to the hospital we get glimpses of fresh green trees in the grounds and grass too, wonderfully bright-looking on a summer day and in such surprisingly close proximity to the great railway terminus. There is a pleasant old-world flavour about the inner precints of Guy's, and its broad-paved foot-ways and wide open spaces give an agreeable impression of a great wealth of spare space. In fact, there is so much ground unappropriated by buildings, that the visitor is somewhat puzzled to understand why ward-kitchens and lavatories should be so cramped for inches, let alone yards of area which might have added considerably to their utility, and to their healthfulness. The "eternal fitness" of things seems somehow absent in these arrangements, and the modern ideas of sanitation teach us to put hygiene even before ornament. The picturesque blocks of the old buildings show that once upon a time land was apparently of no high value hereabouts and they were therefore erected at intervals allowing for plenty of currents of air, and light, and sunshine to penetrate, so far as the fogs of London permit. We cannot but wish that a substantial new nursing home could be erected, as well as the ornate new block for the dental department, which is gradually taking shape. Without doubt the dental branch at Guy's is a big affair, for in mounting to the nurses' quarters we pass a door which opens into a ward, where no less than 90 "chairs" are arranged, all devoted to the dentist's art, and the sight of them gives us a kind of sympathetic toothache as we meditate on the concentration of pain and discomfort expressed by those characteristic preparations!

The nurses' dining-hall is a very fine room, and access to it is gained through the kitchen, which is another relic of by-gone days and ways, we suppose; but the custom which places the paying probationers at a separate table from the other workers, although they feed simultaneously, is one which cannot claim antiquity as its excuse. An underground corridor connects the hall with the hospital, obviating the necessity for nurses to cross the grounds in bad weather. The sisters' meals are served in a room near the dining-hall, and it is well adapted for the purpose, although it does not possess the picturesqueness of the larger apartment. The paying probationers live in the Matron's house, in large airy rooms generally containing two beds, although the occupants of the smaller sized rooms have the advantage of one to themselves. The ordinary probationers and the staff nurses are housed in cubicles situated at the top of one of the blocks of medical wards; they are light and airy, and warmed with hot-water pipes in winter. The night nurses are similarly quartered, but in distinct dormitories quite shut off from the other parts of the building, and at the foot of the staircase which leads to them a door is provided with a silent spring hinge, which seems to act very satisfactorily. There are two sitting rooms for the nurses, and their bookcases contain a good supply of standard works; but, although they are lofty and light apartments, they are somewhat dreary in point of furniture, and also from the absence of any of those pleasant home-like fittings and ornaments with which we have grown familiar in most of the modern nurses' homes. A cottage piano is the only indication of entertainment or relaxation. Unfortunately, these recreation-rooms are placed at a considerable distance from the sleeping quarters, and this must be a drawback to their utility, even though a lift is in constant use for conveying the nurses to and from their cubicles, which is certainly a merciful arrangement for weary feet.

### Nurses' Bookshelf.

"Practical Sanitation,"* by George Reid, M.D., D.P.H., is a well-chosen title for a most useful book. It contains numerous excellent diagrams and an appendix on Sanitary Law, by Herbert Mauley, M.A., M.B., D P.H., which considerably increase the value of the work. Dr. Parkes' practical teaching "that nothing is so costly in all ways as disease, and that nothing is so remunerative as the outlay that augments health, and in doing so augments the amount and value of the work done," is dwelt upon in the preface, and such words of wisdom cannot be too often repeated. Dr. Reid gives some capital advice to Inspectors, and urges them to be perfectly confident of their ground before they condemn existing and satisfactory appliances which are unfamiliar to them. He teaches, in fact, a comprehensive administration as well as a strict one. Information on the subject of hard and soft water supplies, cisterns, domestic filters, sewage, basements, &c., is given in a simple and clear manner, and the chapters on Ventilation and Infection are admirable This book will be useful to all who appreciate the importance of sanitation.

"A Text-Book of Nursing,"† compiled by Clara S. Weeks-Shaw, was first presented to the public in 1885, and the second edition, just issued, is revised and enlarged, with illustrations. It purports to be "for the use of Training Schools, Families, and Private Students," and certainly to all these it will be of substantial value. The volume is of such moderate dimensions that we are surprised to find the amount of information which has been condensed into some four hundred pages. Instruction is given so clearly, and the subjects are so ably arranged and explained that no person can study this little text-book without profit. Miss Weeks-Shaw's definition of nursing and nurses comes home to us all in such sentences as the following: "Unimpaired health and power of endurance, intelligence and common sense, are primary essentials for a nurse. She should be a person of even, cheerful temperament, not easily irritated or confused. . . ." And again, "Loyalty to the doctor includes encouragement of the patient's faith in him, so long as he is in charge of the case." There are many other terse sayings which we have not space to give, but we warmly advise every thoughtful and intelligent nurse to get the book for herself and to study it carefully. Besides the valuable chapters on practical nursing there is a capital list of "Drugs in Common Use, Effects to be Looked for, and Remarks," which is exceedingly useful for all persons trusted with the administration of medicine. Contagion and disinfection are well dealt with, and the subjects are accompanied by some plain remarks, which apply specially to the reader's own common sense. The disinfection of a room by means of sulphur is admirably explained in a short paragraph, and "Emergencies" include poisons and their antidotes, as well as the treatment of broken bones, until the doctor comes. Obstetrics and Sick Children, Convalescence and Death, are written of with thoughtful consideration. The beginning and the end of life are alike comprehensively treated. That important subject, "Food and its Administration," has a special chapter, and various simple recipes for sick diets are given. There is also a handy little vocabulary, before the index, with which closes one of the best nursing books which has ever come into our hands.

* "Practical Sanitation," by George Reid, M.D., D P.H., with Appendix on Sanitary Law by Herbert Manley, M.A., M.B., D.P.H. (Publisher: Charles Griffin, Exeter Street, Strand.)

† "Text Book of Nursing," by Clara S. Weeks-Shaw" (Publishers: D. Appleton and Co., New York.)

### Home for the Dying, Friedenheim.

We gave an account in The Hospital of June 13th, 1891, of the little Home bearing this most touching title. At that time it had already a good record of useful work, for the ten beds appropriated for incurable patients were always full. It is now about seven years since Miss Davidson first carried out her scheme of providing "a quiet place to die in" for the homeless and lonely whose illnesses are of a nature unsuited for prolonged hospital treatment. Many persons have to be discharged from general and special hospitals when their diseases become chronic. The unfortunate sufferers may linger on for months; but as the hospital beds are needed for urgent, hopeful cases, it is neither proper nor desirable to keep them filled with people who cannot derive permanent benefit. Miss Davidson has now taken a new house, which will accommodate four times as many people as her old one. It is "Sunnyside, Upper Avenue Road, South Hampstead, close to Swiss Cottage Station," and it stands in a lovely garden. The rooms are large, bright, and airy, and the sanitary arrangements excellent. The alterations and additions to the house have been planned with practical forethought, which should ensure successful work in all departments. A conservatory provides those patients who can be moved into it with a winter garden in which men obtaining the doctor's permission can enjoy their coveted pipes. The nurses' quarters appear pleasant ones, and the night nurses' rooms are in a block apart from the main building. The Duchess of Teck and Princess May will formally open the Home early in November, when it is hoped that the £10,000 needed for efficient starting of the enterprise will be made up. Already £6,000 have been paid in, and the lease secured for fifty years.

### Everybody's Opinion.

*[Correspondence on all subjects is invited, but we cannot in any way be responsible for the opinions expressed by our correspondents. No communications can be entertained if the name and address of the correspondent is not given, or unless one side of the paper only be written on.]*

### NURSES' CO-OPERATION.

"Nurse M. A. C." writes from 14, Westbourne Place, Clifton, Bristol: Thank you very much for what is in The Hospital paper about the Nurses' Co-operation. The wording was very nice. My fellow nurses are very pleased, and thank you too. Miss Rogers was very surprised and pleased.

### METROPOLITAN DISTRICT SCHOOLS.

A "Would-be Reformer" writes: Dear Sir,—Would it be possible for a representative of The Hospital to visit the Metropolitan District Schools connected with the London workhouses, and to write accounts of the same in a similar way to those of County Hospitals. I am inclined to think that some good might be done in this way towards the improvement of sick wards and sanitation.

### THE WORCESTER INFIRMARY.

"The Nurses of the Worcester Infirmary," under date of September 28th, write: It seems hardly fair that the whole conduct of a matron should be condemned because a probationer who, according to her own admission, had left two hospitals previously, and in each case had spoken slightingly of the matrons, ran off in resentment at fancied ill-treatment. As to Miss Belsey's capabilities, everyone with any hospital experience knows that something more than a refined and gentle spirit is needed to make a good nurse, and that faults which may seem excusable and even lovable in a home circle may need putting down strongly if a woman is to do a work with such responsibility attached as is the case in the nursing profession. With regard to Miss Belsey's not having been off duty for a single day in seven months, owing to sickness, the majority of probationers can say the same, it being an understood thing in most hospitals that probationers shall begin training in a good state of health. Had it been necessary, we know from personal experience that Miss McLelland never grudges a rest for any one ill, and not only that, but is most kind and attentive to anyone suffering, never relegating the duty to others as so many matrons do, but seeing personally that any ailing nurse or probationer is properly treated. If you will kindly find room for this in your valuable paper you will greatly oblige.

### Appointments.

Dundee Convalescent Home.—Miss Jeanie Bowman, who was trained at Dundee Infirmary, has been made Matron of the Dundee Convalescent Home at Broughty Ferry. Miss Bowman was head nurse at Kilmarnock Infirmary (where her sister is Matron) for two years, but returned to Dundee Infirmary in 1887, and worked there until promoted to her present post.

Meath Home for Epileptics.—Miss Anderson, who has been appointed Lady Superintendent of the Meath Home for Epileptics, was trained at St. Bartholomew's Hospital, and afterwards held the post of Night Superintendent at St. Mary's Hospital, where she is much regretted.

Ulverston and District Cottage Hospital.—Miss Jane Huddleston has been appointed Matron of this hospital. Miss Huddleston was trained at the Manchester Infirmary and at Edinburgh. Miss Huddleston has had considerable experience at the Douglas (Isle of Man) Infirmary, and at Dulverton, Somerset. She has been for the last eighteen months on the staff of the British District Nurses' Society.

### Where to Go.

Trained Nurses' Club, 12, Buckingham Street, Strand.—October 14th, at a quarter to eight, Dr. A. Leith Napier will lecture. Subject, "Some Recent Suggestions on Midwifery."

### Notes and Queries.

### Queries.

(1) *Red Cross Sisters; also Army and Navy Nursing.*—Where can I get information about these?—*Nurse G.*

(2) *Nurses' Co-operation.*—Kindly give me the London address?—*Stella.*

(3) *Imbecile.*—Can anyone give address of a suitable home for an imbecile boy of twelve. Parents cannot pay the sum needed at Earlswood, and would be grateful for advice.—*Lambeth.*

(4) *Delirium.*—Will someone kindly suggest the best form of restraining sheet for a severe case of delirium?—*Darlington.*

(5) *Holiday Home, Eastbourne.*—Address wanted for a nurse. *Nurse Bee.*

(6) *Home for Cripple.*—Can anyone suggest home for a young man of 18, who has abscesses on his feet, which keep him in bed for a week at a time. *M. S.*

(7) *Agnes Earl.*—Can any reader oblige Miss Hill, Children's Home, Witney, Oxon., with present address of this lady.—*Miss Hill.*

(8) *Work.*—Will any hospital accept my services (free) from 2 to 7 daily.—*Charlotte.*

### Answers.

(1) *Red Cross, &c. (Nurse G.)*—You will find the information you want in "How to Become a Nurse," by Honnor Morten, price 2s. 6d., Scientific Press, 140, Strand.

(2) *Nurses' Co-operation (Stella).*—Lady Superintendent, 8, New Cavendish Street.

(3) *Imbecile Boy (Lambeth).*—Have you written to Darenth?

(4) *Delirium (Darlington).*—No sheet or other form of restraint should be used without the express order of the medical man.

(5) *Eastbourne. (Nurse Bee.)*—Miss Hackett, Yatton, Eastbourne, has a quiet, comfortable home, and would receive a nurse at moderate terms.

(6) *Cripple. (M. S.)*—Has lad learnt a trade, and what payment can be made for his maintenance? You do not say whether the doctor considers him incurable, nor give your reason for wishing him to leave infirmary.

(7) *Agnes Earl (Miss Hill).*—Have you written to her last address?

(8) *Work (Charlotte).*—Write to the Lady Superintendent, 2, Maida Vale. She is often glad of useful volunteers, and she is a fully-trained lady herself.

### Wants and Workers.

*Nursing Home, East Molesey, Surrey.*—Will any kind friend give an old piano to the above institution for the use of the patients in the long winter evenings.

### The Result of a Chill.

MONITION OF TWO KINDS OF FEVER (*continued*).

Sybil rose and took a letter from her desk.

"This came yesterday," she said. "It is from Mr. Blatch. I thought I would wait and see if our mysterious visitor had sold the picture before I told you about it. But you had better know the worst."

"Won't he buy them?" asked Hilda, in anxiety.

"No; they are 'not in his line,' he says."

"Sybil! What are we to do?"

"Not to worry, for one thing."

"But the rent?"

"Surely Sir Nicholas will wait for a few weeks?"

"Yes, but supposing your picture does not sell?"

"In that case we must part with some of the china," said Sybil.

"Father's cherished vases," murmured Hilda. "It's the first step of a demoralising career when one begins to sell one's heirlooms."

"I would rather sell something else. I have some utterly useless jewellery—we'll let that go first. But don't let us brood over our present impecuniosity."

"I don't wish to, dear. But, really, I don't see how we are going to live, even in our humble style, unless we get some success soon."

"Haven't we been absolutely free from carking care since we came here?" said Sybil. "Aren't we in absurdly rude health, and isn't the freedom of our lives a splendid compensation for our poverty? Come, I think you are raising a bogey. I am not going to be daunted. We can live on the things in the garden, and I'll ask the boy how to set wires for rabbits, if the worst comes to the worst."

"Potatoes are not an invigorating diet," remarked Hilda.

"Nonsense, the Irish thrive on them. You'll find a score of ways of cooking them in the cookery-book. I prefer other foods, but better stay here and eat potatoes than go back to London and throw ourselves into the vortex."

"We certainly cannot expect our stepfather to greet us with much warmth."

"My dear, I would rather slowly pine here than return ignominiously to town and seek relief from a stranger. We have no claim upon a soul. Let us battle for ourselves."

"I'm afraid I'm not so sanguine as you said," Hilda. "The news from Blatch is terrible. Think of it—months of hard work gone for nothing. It takes all the spirit out of me."

"Of course, it is a fearful disappointment; but repining won't mend matters. Try to paint yourself into a more hopeful humour. How do you like this sky? The horizon last night was quite as red, and above it was sapphire."

A colloquy upon the picture distracted them, and before night they had forgotten, for the nonce, that positive destitution was imminent. The contingency of bankruptcy ceases to greatly harass the hardened Bohemian, but the girls, though habituated to pinched living since the death of Captain Ruthven, were unused to serious privation. Hilda's gloomy anticipation of the grave prospect of accumulated liabilities was not lessened by ambition. She was of the temperament that cannot prosper on mere hopes. While Sybil slept, oblivious of impending trials, Hilda lay wide awake in one of those direful midnight moods of apprehension that shorten life. What, after all, were nature, art, health, and youth when the larder was almost empty, and the bunch of bills behind the sitting-room mirror was growing bulky? To be unable to pay cash down for everything seemed to her an ill that had no amends in beautiful surroundings. She wished she could share her sister's sustenance in the rapt study of sunsets. When she came back from the downs with a cheerful wind-reddened face, and the appetite of a sportsman, Sybil spoke of nature as a kind of charm, an effectual panacea for all mental pangs, and she would relate the trivial sights and incidents of the day with the zest of a true monomaniac of fresh air. A walk to the top of the Beacon, fourteen hundred feet above the sea, dispelled her cares like strong wine.

"I begin to think Mr. Lowther is not acting in a business-like manner," said Hilda, one day. "You ought to write to him."

"What do you expect him to do?" asked Sybil.

"To tell us if the picture is sold."

"It isn't, or we should have had the money. We must have patience."

"Why have you such implicit faith in him?"

"Because he is undoubtedly a man of honour. One may read that in his face."

"Really, Sybil, you are amazingly confident of this utter stranger. For my part, I begin to distrust his plausible manner, and to suspect him for an adventurer."

"A plunderer of two defenceless maidens, I suppose," laughed Sybil. "I have a less pessimistic view of human nature."

Slightly wearied with Sybil's seeming indifference to their future fate, Hilda at length determined to unravel the mystery of F. Lowther. It would be easy to find out if the stranger was a picture dealer. Her old friend and school-fellow, Kate Masters, would call at the shop, and see if the proprietor's personal appearance corresponded with her description of him. So without mentioning the matter to her sister, Hilda wrote to her friend, posting the letter when she went to Chagford. In three days an answer came. Kate Masters had been to the shop and discovered that F. Lowther was a very old dusty gentleman, who dressed in obsolete garb, and took snuff. She said the place was small and untidy, and it looked as though very little business was done there. When Hilda read the letter, Sybil's lips twitched with indignation.

"This is all very repugnant to me," she said, flushing. "I don't know why you have elected to play the spy upon one who is evidently anxious to act as a friend. I never thought him a picture dealer. It was you who suggested it first. What good has your action done?"

"It has convinced me that we ought to pursue investigations," replied Hilda. "Still, if you choose to let your picture be stolen, that is your affair. I am glad I did not entrust the man with one of mine."

*(To be continued.)*

### Hints for Nurses.

We have just seen some patterns of excellent serges from the manufactory of Messrs. Pease, of Darlington, which appear especially suitable for nurses' wear. The "I.D. Melton Cloth" would make an excellent winter dress, and it is a wide width, and moderate in price. The "I.D. Union Fonté" is also very tempting, and is somewhat thinner in texture and brighter in colour, but both are good navy blues. Messrs. Pease have also many light woollen materials which would wash well, and are, therefore, suited for private nurses' uniform dresses.

## Figures and Tables

**Figure f1:**